# Mortality and life expectancy forecast for (comparatively) high mortality countries

**DOI:** 10.1186/s41118-018-0042-x

**Published:** 2018-11-01

**Authors:** Ahbab Mohammad Fazle Rabbi, Stefano Mazzuco

**Affiliations:** Department of Statistical Sciences, University of Padua, Via Cesare Battisti 241, Padua, 35121 Italy

**Keywords:** Mortality forecast, Lee–Carter method, Coherent forecasting, Mortality in Eastern Europe, Bayesian hierarchical model

## Abstract

**Background:**

The Lee–Carter method and its later variants are widely accepted extrapolative methods for forecasting mortality and life expectancy in industrial countries due to their simplicity and availability of high quality long time series data.

**Objective:**

We compared and contrasted mortality forecasting models for higher mortality regimes that lack long time series data of good quality, which is common in several Central and Eastern European (CEE) countries.

**Data and methods:**

We utilized seven different variants of the Lee–Carter method and coherent mortality forecasts of various CEE countries, and the Bayesian Hierarchical Model used by the United Nations to produce probabilistic forecasts. The data of nine CEE countries with comparatively higher mortality have been considered.

**Results:**

The performance of the forecasting models for the nine CEE countries was found to be lower than that observed for low-mortality countries. No model gives uniquely best performance for all the nine CEE countries. Most of the LC variants produced lower forecasts of life expectancies than current life expectancy values for Belarus, Russia, and Ukraine. A coherent mortality forecast could not overcome the limitations of single population forecasting techniques due to increasing mortality differences between these countries over the fitting period (mortality divergence). In the same context, the use of the probabilistic forecasting technique from the Bayesian framework resulted in a better forecast than some of the extrapolative methods but also produced a wider prediction interval for several countries. The more detailed analysis for Hungary indicates that a better fit of certain forecasting methods may occur in the later part of the life span rather than the whole life span.

**Conclusion:**

These findings imply the necessity of inventing a new forecasting technique for high-mortality countries.

## Introduction

Improved mortality has been observed globally during the twentieth century and is always considered a positive change for the socio-economic advancement of a country. This change has brought new requirements to support systems for the elderly, such as improved health care and pension provision. Aging has become the greatest population problem for many industrialized countries since the 1970s (Brouhns et al. 2002). This has resulted in increasing interest among government policy makers and planners in accurately modeling and forecasting age-specific mortality rates. Policymakers rely greatly on the possibility of forecasting future population structures. Population aging is increasing not only in low-mortality industrialized countries but also in several Eastern European countries including Russia. These countries have a slower pace of mortality improvement in several stages of the life span compared to low-mortality countries, which delayed the aging problem (Gavrilova and Gavrilov 2009). This leads to the necessity for research on mortality forecasting from the perspective of these countries.

Several parametric and nonparametric methods have been proposed over the years in order to forecast age-specific mortality rates and life expectancy. Booth and Tickle (2008) reviewed all existing mortality forecasting methods under three broad classes in terms of expectation, extrapolation, and explanation. The simplest way of parametric forecasting is to parameterize the available series of life tables and extrapolate each of the parameters separately to obtain the forecast using the assumed model (Keyfitz 1991). In addition to all subjective approaches, a groundbreaking approach to probabilistic forecasting was proposed by Lee and Carter (1992). The advantages of the Lee–Carter (LC) method are its simplicity, as it has few parameters and a straightforward explanation, and robustness in situations where age-specific log mortality rates have linear trends (Booth et al. 2002). To increase the precision of the LC method in the presence of an irregular mortality schedule, later studies restricted the fitting period to post-war years, along with other modifications (Lee and Miller 2001). More generally, Booth et al. (2002) noticed the length of the fitting period might greatly affect point forecast accuracy. Later nonparametric methods of mortality forecasting were introduced by Hyndman and Ullah (2007) and were based on the LC framework. Nonparametric methods were found to be more robust, as they are more efficient than other LC variants, even in presence of outliers, and provide more accurate forecasts (Shang et al. 2011). Numerous other methods have been proposed for mortality forecasting, although many are extensions of the basic LC method. Renshaw and Haberman (2000) used a generalized linear model to model mortality reduction factors and identified the conditions under which the underlying structure of the proposed model is identical to that of the LC method. A further extension by Renshaw and Haberman (2003) accounts for the cohort effect. Other stochastic models have also been introduced to integrate the cohort dimension in mortality, for example that by Cairns et al. (2011). In addition to several LC variants, the application of the Bayesian framework to the LC methodology has become popular. A few studies have proposed extensions of the LC method using a Bayesian framework (see Wiśniowski et al. 2015, for example). Mortality forecasts might also be affected by a different distribution of causes of death across countries (see Booth and Tickle 2008) and risk factors (see Janssen et al. 2013, for an example on smoking status). De Beer (2012) introduced a new relational model for both smoothing and projecting age-specific mortality rates. Mortality forecasting based on the distribution of death has recently become more popular. These approaches are gaining traction because they are independent of the LC framework and are able to overcome some of the basic limitations of the LC methodology (De Beer et al. 2017). Nevertheless, these approaches are newer, and LC variants are the most applied techniques for mortality forecasting (Bohk-Ewald and Rau 2017).

All these methods predict the future mortality of a single population without considering impact of neighbor countries (geopolitically, socio-economically, or by any other common criteria) which may significantly impact the mortality of that population (Hyndman et al. 2013). The first approach to coherent mortality forecasting was introduced by Li and Lee (2005) as an extended hierarchical interface of the LC method. Later, Hyndman et al. (2013) extended the nonparametric approach of Hyndman and Ullah (2007) for coherent forecasting; Ahmadi and Li (2014) used generalized linear modeling and Bergeron-Boucher et al. (2017) used compositional data analysis on distribution of death for coherent forecasting. In another approach, Janssen et al. (2013) considered the smoking epidemic for coherent forecasting.

Given the existence of so many forecasting methods, it is particularly important to assess which model is the most useful in specific contexts. Such an assessment has been conducted by Shang et al. (2011), even though only a specific context was considered, that is, industrialized countries characterized by low mortality, high life expectancies, lower adult and early senescence mortality, a stable pattern of mortality transition over time, and high data quality. Similarly, only low-mortality, industrialized countries have typically been considered in coherent forecasting (see Seligman et al. 2016, for example). Fewer attempts have been made to consider high-mortality Central and Eastern European (CEE) countries or a large number of different mortality forecasting methods (see Bohk and Rau 2015, for example). These countries were also mentioned before for increasing mortality differences over time (the divergence of mortality regime), whereas the developed countries in Western Europe have almost opposite scenario (Vallin and Meslé 2001; Gavrilova and Gavrilov 2009). Even though the mortality pattern is still different from that of Western European countries (see Vallin and Meslé 2001), there are also some similarities (Bálint and Kovács 2015), including increasing population aging (see Gavrilova and Gavrilov 2009). Due to limitations of long time series data (with good quality), it is possible that high-mortality regime forecasts will not be the same as those obtained by Shang et al. (2011) or Shang (2012). This expectation is somewhat confirmed by Bohk and Rau (2015) who compared and contrasted forecasting techniques to evaluate the impact of the recent financial crisis on some of the CEE countries and stated that irregular mortality developments are particularly difficult to forecast due to major changes in long-term trends. Therefore, the CEE countries would benefit from an accurate mortality forecasting by comparing the outcomes of different forecasting techniques.

The aim of this paper is to assess the performance of selected forecasting methods for countries characterized by a higher mortality regime vis-$\grave {\mathrm {a}}$-vis Western countries. We compared and contrasted the performance of the mortality forecasting models for nine CEE countries—Belarus, Bulgaria, Estonia, Hungary, Latvia, Lithuania, Russia, Slovakia, and Ukraine. The selected CEE countries differ from Western European and other non-Eastern European countries in five ways: (a) higher mortality, (b) irregular mortality trends, (c) increasing mortality differences between countries over time (the divergence of mortality regime), (d) shorter time series data, and (e) lower quality mortality data. Following Shang et al. (2011), we limited our main interest on LC variants. As mentioned, LC variants are widely used because of their simplicity; many European countries use LC variants for official forecasts (Stoeldraijer et al. 2013). Basic LC method is the main one used for comparisons due to its widespread acceptability (see Booth et al. 2012, Hyndman and Ullah 2007, Shang 2002, for example). Moreover, we considered two more approaches for comparison—coherent mortality forecasting and life expectancy forecasting using a Bayesian Hierarchical model adapted by the United Nations (UN). We extended this comparison to a coherent setup because on contrary to the concept of coherent mortality forecasting, these countries have diverging mortality patterns compared to those of low-mortality countries (Li and Lee 2005). Thus, this extension can provide more insight regarding the assessment of coherent mortality forecasting. The UN adapted the probabilistic approach for forecasting life expectancy using a Bayesian hierarchical model over the previously used deterministic approach (Raftery et al. 2013). UN projections are widely accepted even for countries with limited data, therefore, we extended our comparison to include this technique. Overall, this sort of comparison of mortality forecasting for high-mortality countries may help researchers to better understand the scope of developing mortality forecasting models and may help policymakers in terms of policy implications relating to age- and cause-specific mortality. Unlike the study by Shang (2012), comparing and contrasting on the abovementioned comparatively high mortality countries may give us better insight regarding the impact of recent mortality improvements on future mortality.

This paper consists of four parts. In the next section, we review the forecasting models used in this study and the source of data. The “Results” section consists of two subsections: in the first one, we compared the findings of the models for selected countries with high-mortality regimes, while in the second one, we discuss the country-specific results for Hungary to gain more insight regarding the fitted models. The last section consists of the concluding remarks.

## Data and methodology

### Data

The data used in this study came from the Human Mortality Database (HMD 2018). Data from nine CEE countries are utilized. Central and Eastern Europe (including Russia) has higher mortality compared to Western Europe. These countries were mentioned before for increasing mortality differences between countries over time (the divergence of mortality regime), whereas the developed countries in Western Europe have converging trends (Vallin and Meslé 2001). Some of these former Socialist countries have some similarities in terms of social inequalities (Mackenbach 2013). We excluded Poland and Slovenia from this analysis as their mortality patterns are more similar to those in Western European countries. The data we used for different countries and their last observed life expectancies at birth are given in Table 1. For most of these countries, the life table started from 1950; only Bulgaria has available data from 1947. However, we did not utilize all the available data for several of these countries due to the lower quality of the data, as mentioned by the HMD (HMD 2018).

**Table 1:** Fitting periods for the countries and life expectancy at birth (HMD 2018)

Country	Starting year	End year	*e*_0_ (female)	*e*_0_ (male)
Belarus	1970	2014	78.43	67.81
Bulgaria	1950	2010	77.25	70.31
Estonia	1959	2013	81.33	72.72
Hungary	1960	2014	79.24	72.26
Latvia	1970	2013	78.73	69.26
Lithuania	1959	2013	79.37	68.52
Russia	1970	2014	76.48	65.26
Slovakia	1962	2014	80.32	73.25
Ukraine	1970	2013	76.21	66.31

For the Bayesian probabilistic model (UN forecast), the life expectancy at birth data are taken from the HMD on a 5-year basis rather than complete life tables used for the extrapolative models. We also contrast this method using the data of World Population Prospects 2012 (UN 2013).

### Mortality projection models

The models used in this study are briefly reviewed in the following subsections. We used seven different variants of the LC method along with a coherent mortality forecasting method and the Bayesian hierarchical model used by the UN to produce probabilistic forecasts. We considered four parametric LC variants those by Lee and Carter (1992), Lee and Miller (2001), Booth et al. (2002), and Brouhns et al. (2002), and three nonparametric variants of the Hyndman–Ullah method, robust Hyndman–Ullah method, and weighted Hyndman–Ullah method (Hyndman and Ullah 2007).

#### Lee–Carter method (1992) and variants

Since its development, the LC method has been one of the most applicable methods. Use of principal components for mortality forecasting came to practice through the work of Lee and Carter (1992). The two-factor LC model is given below:

1 $$ \ln m_{x,t}=a_{x}+b_{x}k_{t}+\epsilon_{x,t}.  $$

Here, *m*_*x*,*t*_ is the central mortality rate at age *x* for year *t*, *a*_*x*_ represents the average log mortality at age *x* over time, *b*_*x*_ is the first principal component capturing relative change in the log mortality rate at each age *x*, *k*_*t*_ represents the overall level of mortality in year *t*, and *e*_*x*,*t*_ is the model residual. Singular value decomposition (SVD) is applied to $Z_{x,t}=\![\!\ln (m_{x,t})-\hat {a}_{x}]$ to obtain the ordinary least squares (OLS) estimate of the LC model. SVD decomposes the *Z*_*x*,*t*_ into the product of three matrices. Symbolically,

$$\text{SVD} (Z_{x,t})=ULV^{\prime}=L_{1}U_{x_{1}}V_{t_{1}}+\ldots L_{n}U_{x_{n}}V_{t_{n}}. $$

To estimate of the age and time components, Lee and Carter (1992) considered the rank-1 approximation only because it explains most of the variance. The estimates of the model parameters are:

$$\hat{k}_{t}=L_{1}V_{t_{1}} \text{and }\hat{b}_{x}=U_{x_{1}}. $$

The LC method makes a second stage estimate of *k*_*t*_ by finding the value of *k*_*t*_, which for a given population age distribution and previously estimated *a*_*x*_ and *b*_*x*_ produces exactly the observed number of total deaths for the fitting period of the model (Lee and Carter 1992). An *ARIMA(0,1,0)* with drift is then fitted for adjusted $\hat {k}_{t}$ and used to forecast future mortality. Later, Lee and Miller (2001) proposed three modifications of the basic LC model: (i) the fitting period is restricted from 1950 and onward to reduce structural shifts, (ii) the adjustment of *k*_*t*_ is done by matching life expectancy, and (iii) the “jump-off error” is eliminated by forecasting forward from observed (rather than fitted) rates.

The LC model can be extended by including higher-order terms instead of the rank-1 approximation considered in the earlier two approaches. Higher-order terms were modeled by Booth et al. (2002), and forecasts were later developed using univariate ARIMA processes (Renshaw and Haberman 2003). The key modifications of this method are (i) the fitting period is determined by a statistical “goodness of fit” criterion, under the assumption that *k*_*t*_ is linear; and (ii) the adjustment of *k*_*t*_ involves fitting to the age distribution of deaths rather than to the total number of deaths in the basic LC model. This model is a significant development in the research of forecasting mortality, as it slightly eliminates a shortcoming of the LC model. The LC model assumes invariant *b*_*x*_, whereas evidence of substantial age–time interaction is common (Shang 2012).

Brouhns et al. (2002) considered the underlying deaths are distributed in a Poisson regression and assumed them to have the following log-bilinear form of the mortality rates.

$$D_{x,t}\sim \text{Poisson}\left\lbrace E_{x,t}m_{x,t}\right\rbrace \text{ with }m_{x,t}=\exp(a_{x}+b_{x}k_{t}); $$

where *E*_*x*,*t*_ represent the population exposed to death at age *x* in time *t*. The constraints of the basic LC model also holds for this method. One of the main advantages of using this approach is that it allows the maximum likelihood estimation of the model parameters instead of OLS or a Gauss–Newton algorithm (Brouhns et al. 2002). This shows some further development in scope to utilize the Bayesian approach for LC methods. In this paper, our analysis was based on the classical approach instead of the Bayesian framework.

### Nonparametric approaches: the Hyndman and Ullah (2007) methods

To address the problem of lack of across-age smoothness, heterogeneity of deaths over a long time period (Girosi and King 2006) and the consideration of only the first principal component in LC variants, Hyndman and Ullah (2007) proposed a functional data model that utilizes second- and higher-order principal components to capture additional variation in mortality rates. This technique uses a penalized regression spline with a partial monotonic constraint to smooth the log mortality rates first. The following continuous smooth function *f*_*t*_(*x*) is assumed for discrete ages.

2 $$ m_{t}(x_{i})=f_{t}(x_{i})+\sigma_{t}(x_{i})\epsilon_{t,i};\quad i=1,\ldots,p;\quad t=1,\ldots,n;  $$

where *m*_*t*_(*x*_*i*_) represents log-transformed mortality rates for each age *x*_*i*_ in time *t*, *σ*_*t*_(*x*_*i*_) is the noise component and *ε*_*t*,*i*_ is an i.i.d. standard normal variable. Hyndman and Ullah (2007) proposed to use weighted penalized regression splines to estimate *f*_*t*_(*x*). This weighting controls heterogeneity due to *σ*_*t*_(*x*), and a monotonic constraint for upper ages can lead to better estimates. In this study, we applied equal weights to the approximate inverse variances *w*_*x*,*t*_=*m*_*x*,*t*_*E*_*x*,*t*_ and used weighted penalized regression splines to estimate the curve *f*_*t*_(*x*) for each year (Hyndman and Ullah 2007). Weighted penalized regression splines are preferable in terms of computational time and allow monotonicity constraints (Hyndman and Ullah 2007). Details of the estimation procedure for interval forecasts are given elsewhere (Hyndman and Ullah 2007). Functional principal component analysis utilizes a set of continuous functions and is decomposed into functional principal components and their associated scores, symbolically

$$f_{t}(x)=a(x)+\sum\limits_{j=1}^{J}b_{j}(x)k_{t,j}+e_{t}(x); \quad t=1,\ldots,n; $$

where *a*(*x*) is the mean function $\left (=\frac {1}{n}\sum _{t=1}^{n}f_{t}(x)\right)$, *b*_*j*_(*x*) is the set of first *J* functional principal components, *k*_*t*,*j*_ is the set of uncorrelated principal component scores, and *e*_*t*_(*x*) is the residual function. It should be noted that *J*<*n* is considered for optimal number of functional principal components. The ARIMA model is suggested to forecast principal component scores, as they have minimum AIC (Akaike information criterion) of the fitted model; however, almost every suitable time series can be applied (Shang 2012). Two more versions of the HU method were also proposed for special situations. The first one is generally refereed to as the robust Hyndman and Ullah method (HU_R_), proposed to forecast in presence of outliers. This approach investigates the integrated squared error for each year by calculating following measures of accuracy for the functional principal component approximation of the functional data.

3 $$ \int_{x-1}^{x_{p}}\left(f_{t}(x)-a(x)-\sum\limits_{j=1}^{J}b_{j}(x)k_{t,j}\right)^{2}dx.  $$

After assigning zero weight to outliers, the HU_R_ fits the mortality rates from which forecasts of age-specific life expectancies can be estimated without the effect of prospective outliers. Hyndman and Ullah (2007) proposed another weighted version of HU where recent years get more weight during model fitting than years from the distant past. The new method can be showed symbolically as follows:

4 $$ f_{t}(x)=\widehat{a}^{*}(x)+\sum\limits_{j=1}^{J}b_{j}^{*}(x)k_{t,j}+e_{t}(x);  $$

where, *a*^∗^(*x*) is the weighted functional mean, such as:

$$\widehat{a}^{*}(x)=\sum\limits_{t=1}^{n}w_{t}f_{t}(x),\quad \sum\limits_{t=1}^{n}w_{t}=1, \text{ where,}\ w_{t}=\kappa(1-\kappa)^{n-t};\quad t=1,\ldots,n. $$

This *w*_*t*_ is the new weight defined by Hyndman and Ullah (2007) for 0<*κ*<1, a geometrically decaying weight parameter. The optimal value of *κ* is chosen by minimizing an overall forecast error measure within the validation data set among a set of possible candidates. Details of the methods can be found elsewhere (Hyndman and Shang 2009).

### Coherent mortality forecasting

In recent years, coherent or multi-population forecasting methods have become more popular because these approaches try to capture the effects of global improvements in health, communication, and science on a specific population. The standard (Lee and Carter 1992) model and its variants are designed for forecasting for single populations and are often used for females and males independently. Li and Lee (2005) modified the standard LC model to forecast mortality for countries by taking into account their membership in a group, rather than forecasting individually. To do that, Li and Lee (2005) first identified the central tendencies within a group, addressing as common factor and hence adopted the historical particularities of each country as their due weight in projecting individual country trends for forecasting mortality. Thus, in the short term, inter-country mortality differences in trends may be preserved, but ultimately age-specific death rates within the group of countries are constrained to maintain a constant ratio to one another (Li and Lee 2005). This extended model can be formulated as:

5 $$ \ln m_{x,t,i}=a_{x,i}+B_{x}K_{t}+b_{x,i}k_{t,i}+\epsilon_{x,t,i};  $$

where *i* stands for a specific country in the group, and *a*_*x*,*i*_ is the country-specific average log mortality rate. The terms *B*_*x*_ and *K*_*t*_ are relative speed of change in mortality at each age *x* and a mortality index capturing the main time trend for the reference population, respectively. Li and Lee (2005) mentioned the term *B*_*x*_*K*_*t*_ as a common factor because this quantity is common for all the countries of the group. The error term of Eq. (5) is the country-specific estimate of the error. To obtain the country-specific estimates of the Li–Lee model, first the Lee and Miller (2001) model is fitted to a reference population. The reference group is constructed by adding all the populations of the group from which the common factor is extracted to use in the country-level mortality forecasting mentioned in Eq. (5). Both for *K*_*t*_ and *k*_*t*,*i*_, random walk with drift is used for forecasting in the current study. Following Lee and Miller (2001), actual data is used for mortality forecasting (rather than fitted data) to avoid jump-off error. Choosing a reference population remains one of the biggest problem in coherent forecasting. Several approaches have tried different strategies for using particular countries as reference populations considering geographic and economic similarities, and other criteria (Kjærgaard et al. 2016). In the current study, we combined all populations together as a reference group for each of these countries. Li and Lee (2005) also considered a group of low-mortality countries as a reference for making coherent forecasts for high-mortality countries with the optimistic assumption that these countries will catch-up with the low-mortality countries in future.

Besides these abovementioned variants of LC and HU method, there are other approaches for mortality forecasting. Most of these techniques are largely based on the original LC method. For example, Tuljaparker et al. (2000) used the LC model without any adjustment of the time component and started model fitting from 1950; Girosi and King (2006) considered more than one component in the LC model, and later in another study, they extended the model to incorporate age–period–cohort effects. Using the parameter estimation technique of Brouhns et al. (2002), several approaches have been proposed on Bayesian framework. For the sake of simplicity and wide applicability, we restrict our comparison to major variants of the LC and HU methods only.

### Bayesian probabilistic projections (UN life expectancy forecast)

Several mortality forecasting techniques using the Bayesian framework have been proposed to overcome invariant mortality improvements in LC variants (e.g., Bohk-Ewald and Rau 2017; Cairns et al. 2011; Wiśniowski et al. 2015). In addition to this methodological problem, one of the major shortcomings of all the abovementioned variants of the LC model is that these methods require age-specific death rates for at least three decades to fit the model, certainly not the case for many developing countries (Raftery et al. 2013). Instead of forecasting mortality rates in Bayesian framework, (Raftery et al. 2013) proposed an alternative approach to forecast life expectancy at birth using the Bayesian framework. They applied a Bayesian hierarchical model to forecast period life expectancy directly using a random walk model with a non-constant drift. The newly defined drift term is a nonlinear function of current life expectancy and reflects the fact that life expectancy tends to change more slowly for the countries with the lowest and highest life expectancies and more quickly for the countries in the middle. The UN produces estimates of age-specific mortality and period life expectancy at birth for all member countries and updates in every 2 years in the UN World Population Prospects (UN 2013). The UN projects life expectancy in the next time period deterministically using the equation:

6 $$ \ell_{c, t+1}=\ell_{c,t}+g(\ell_{c,t}).  $$

Forecasting life expectancy is done using a double logistic function of the current level of life expectancy, symbolically represented as:

7 $$\begin{array}{*{20}l} g\left(\ell_{c,t}|\theta^{c}\right) =& \frac{k^{c}}{1+\exp\left(-\frac{A_{1}}{\Delta_{2}^{c}}\left(\ell_{ct}-\Delta_{1}^{c}-A_{2}\Delta_{2}^{c}\right)\right)}  \\ &+\frac{z^{c}-k^{c}}{1+\exp\left(-\frac{A_{1}}{\Delta_{4}^{c}}\left(\ell_{ct}-\sum_{i=1}^{3}\Delta_{i}^{c}-A_{2}\Delta_{4}^{c}\right)\right)}. \end{array} $$

$\Delta _{1}^{c},\Delta _{2}^{c},\Delta _{3}^{c},\Delta _{4}^{c}, k^{c},$ and *z*^*c*^ are the six parameters of the double logistic function for country *c* at time *t*. The estimation technique has changed since World Population Prospects 2012 (UN 2013), as the UN Population Division used a probabilistic model for the first time to forecast life expectancy at birth using the methods of Raftery et al. (2013). They utilized the following hierarchical model to turn the old deterministic model into a probabilistic one (with uncertainty) and hence adopted a Bayesian approach to estimate the model parameters. Therefore, the hierarchical model become:

8 $$ \ell_{c,t+1}=\ell_{c,t}+g\left(\ell_{c,t}|\theta^{(c)}\right)+\epsilon_{c,t+1}.  $$

Raftery et al. (2013) defined proper prior for all 13 parameters of the model in such a way that the prior distributions are more diffuse than the posterior distributions. Thus, the abovementioned hierarchical model turned into a Bayesian hierarchical model. This method has one advantage over any other parametric or nonparametric methods; it is flexible for choosing the prior to obtain fast, slow, or medium pace of change in level of life expectancy, per the usual UN projection. The UN method has been proposed to forecast life expectancy using data of World Population Prospects or similar sources. As a consequence, one of the major disadvantages of this method is that it does not forecast considering whole life tables like the previous methods. This makes it complicated to compare the outcomes with those of LC variants. This method takes a single value for life expectancy for each of the 5 years and also returns the forecast as a single value for a period of 5 years (as a median life expectancy for 5 years) utilizing a Bayesian hierarchical model. We considered this method for comparison to evaluate whether LC variants are more optimistic than those used in forecasting only life expectancy at birth. None of the previous approaches compared their findings with UN projections.

### Assessing the performance of the mortality forecasting techniques

We assess the performance of the mortality forecasting techniques in general for all the countries using three classes of forecasting techniques: LC and HU variants, coherent forecasts, and UN projections. As coherent forecasting and UN projections are different than LC and HU variants in terms of interpretation and implications, we assess them in different subsections of the “Results” section. The problem of the shortened fitting periods is illustrated in the subsection on coherent forecasting. We fitted the models for 18 series of life tables for males and females separately. The analysis performed in this study are implemented by “Demography” package of R for all of the LC and HU variants. For Bayesian forecast, we used the “bayesLife” package in R. For illustration, we presented the results for females only. To evaluate a forecast technique, we considered two criteria—how optimistic the forecast is in the long run and the accuracy level during the out-of-sample evaluation period (except for the UN method). For high-mortality countries with a jagged pattern of mortality improvement over the years, it is possible that the forecast could be lower than the last observed life expectancy (Booth et al. 2002). We considered it a failure of the model to capture the mortality trend for that particular country because a forecast showing future decline in the mortality pattern is contrary to the basic assumption of the models regarding future mortality improvement (Lee and Miller 2001). Moreover, lower estimates of life expectancy may occur due to a seasonal jump of life expectancy during the out-of-sample evaluation period or in the short run, which is not the case for the long forecast horizon (Shang 2012). Although we have included the UN forecast technique to compare with our findings, we still limit the main topic of discussion to the LC and HU variants.

To estimate the forecast accuracy, we used the mean absolute forecast error (MAE) and mean squared forecast error (MSE), symbolically represented as:

$$\text{MAE}=\frac{1}{(p+1)q}\sum\limits_{j=1}^{q}\sum\limits_{x=0}^{p}\left|y_{x,j}-\widehat{y}_{x,j|j-h}\right|, $$

$$\text{MSE}=\frac{1}{(p+1)q}\sum\limits_{j=1}^{q}\sum\limits_{x=0}^{p}\left(y_{x,j}-\widehat{y}_{x,j|j-h}\right)^{2}. $$

Here, *y*_*x*,*j*_ represents the observed mortality rate for age *x* in year *j*, and $\widehat {y}_{x,j}$ represents the forecast. Unlike Shang et al. (2011) or Shang (2012), we choose the mean squared forecast error over the mean forecast error as a measure of forecast accuracy. Most of the time, the mean forecast error can be misleading because it may conceal forecast inaccuracy due to the offsetting effect of large positive and negative forecast errors or very low error in forecasting (actual). We used available mortality data from the last 10 years for the out-of-sample evaluation of the forecasting technique. Using the data in the fitting period, we made the one-step-ahead and ten-step-ahead point forecasts and determined the forecast accuracy by comparing the forecasts with the holdout data in the out-of-sample evaluation period.

Due to a shorter fitting period for most of these countries, we kept the forecast horizon for 40 years only; this is the approximate available length of the fitting period for all of these countries. For high-mortality countries, this ground also seemed safe to us like the previous studies conducted on low-mortality countries (e.g., Janssen et al. 2013). Here, LC stands for the basic (Lee and Carter 1992); LC_P_ stands for the model with a Poisson regression (Brouhns et al. 2002); LM stands for the modified LC model proposed by Lee and Miller (2001); BMS stands for the modified LC model proposed by Booth et al. (2002); HU stands for the nonparametric approach proposed by Hyndman and Ullah (2007); HU_R_ stands for the robust (Hyndman and Ullah 2007); HU_W_ stands for the weighted (Hyndman and Ullah 2007); LL stands for the coherent mortality forecast proposed by Li and Lee (2005); and UN stands for the UN life expectancy forecast using a Bayesian hierarchical model (Raftery et al. 2013). These notations are used throughout the paper. Except for the coherent forecasting, the fitting periods are the same for the rest of the models (see Table 1). To keep the comparison of models in same the time frame as the Lee and Miller (2001), we started the fitting from 1950. For coherent forecasting, we used the common fitting period of 1970 to 2010 for all the countries. We used the life tables constructed up to age 100 for model fitting and forecasting due to the lack of data for most of the countries at later age groups.

## Results

### Comparison of mortality projection models for the nine selected CEE countries

Forty years ahead point forecasts of life expectancy at birth obtained using all the methods are given in Table 2. In Table 2, forecast of life expectancy is presented only for the models for which the 40 years ahead forecast is higher than the last observed life expectancy. The results of the different types of models are discussed later.

**Table 2:** Comparison of 40 years ahead life expectancy forecast for selected countries with high mortality

Country	*e*_0_	LC	LC_P_	LM	BMS	HU	HU_R_	HU_W_	LL	UN
Belarus	78.43	–	–	78.43	–	–	–	80.79	–	79.95
Bulgaria	77.25	82.16	79.77	80.24	79.76	78.49	80.83	82.61	77.55	80.63
Estonia	81.33	85.98	85.99	86.19	91.00	84.19	82.74	84.95	84.71	85.70
Hungary	79.24	83.16	83.05	83.68	85.98	84.64	82.99	87.81	82.60	83.54
Latvia	78.73	81.87	81.76	81.92	86.37	81.30	81.76	82.31	80.10	82.43
Lithuania	79.37	82.31	81.05	81.93	85.20	80.37	80.09	82.70	81.63	82.76
Russia	76.48	–	–	–	–	–	77.82	–	–	79.08
Slovakia	80.32	84.91	84.65	84.66	86.24	83.93	83.64	84.17	82.37	84.44
Ukraine	76.21	–	–	–	–	–	80.19	78.64	–	79.24

Results are shown for females only.

*e*_0_ is the last observed life expectancy during the fitting period from HMD (Table 1).

A blank place means the forecast of e_0_ was lower than the last observed value of *e*_0_.

#### LC and HU variants

**Fig. 1:**
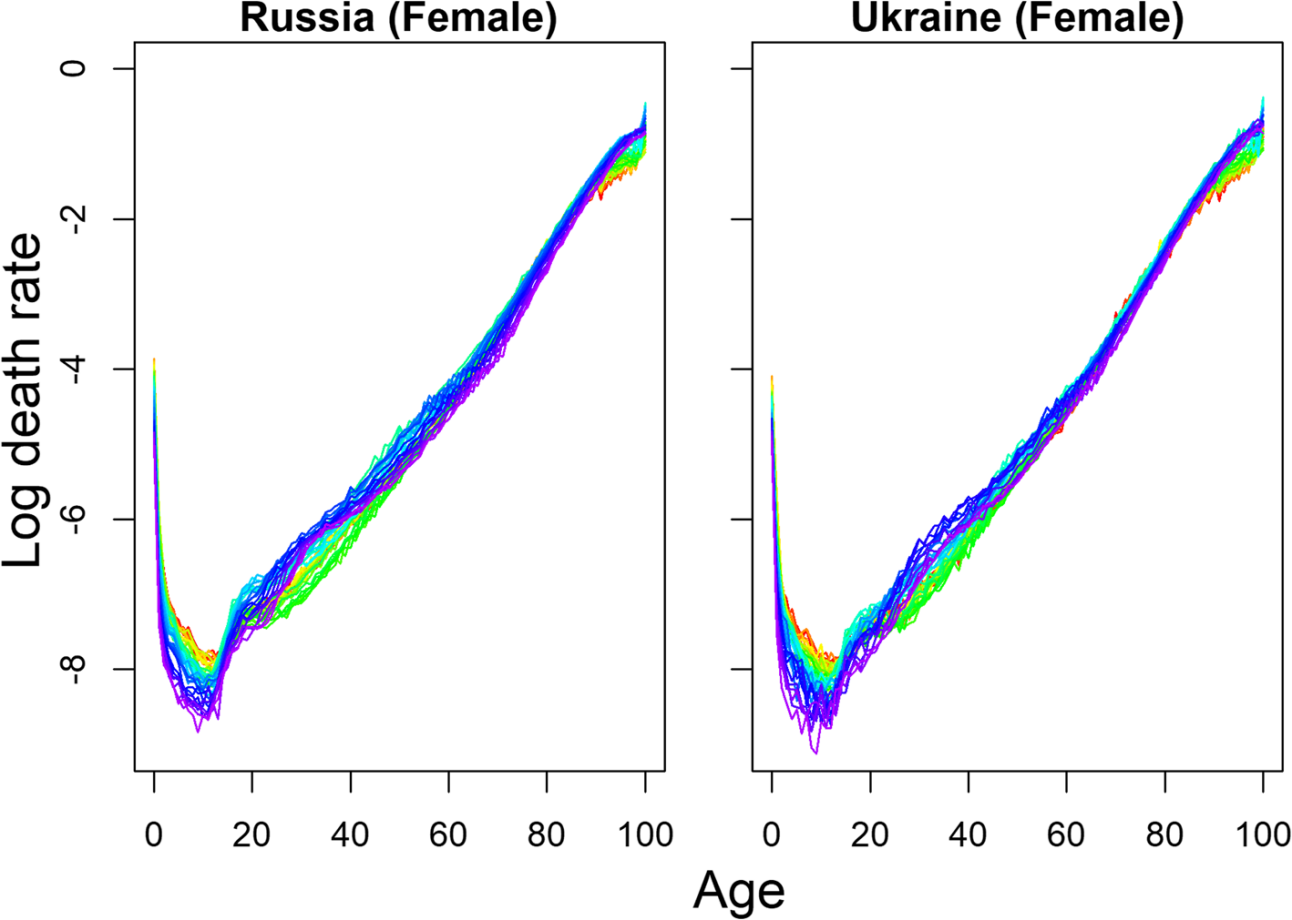
Observed mortality rates of Russia (1970–2014) and Ukraine (1970–2013). Years are plotted using a rainbow palette so the earlier years are shown in red, followed by orange, yellow, green, blue, and indigo with the most recent years plotted in violet

BMS produced the most optimistic forecast of life expectancy for three Baltic countries and Slovakia. HU_R_ produced the highest forecast for the Ukraine; the UN forecast was the highest for Russia, and for rest of the countries, HU_W_ produced the most optimistic point forecast of life expectancy. In addition to other exceptional cases, all the models produced lower forecasts than the last observed life expectancy for Belarus, Russia, and Ukraine, the countries with the highest mortality levels. Only HU_W_ could produce a higher forecast of life expectancy than the last observed *e*_0_ for Belarus; all other methods extrapolated lower or almost equal future life expectancy. For Russia and Ukraine, the HU_R_ method was appropriate in this sense (for Ukraine, HU_W_ was also appropriate). The results of HU variants show a better fit. This is attributable to smoothing and the implications of more than one principal component to explain the higher variation (Hyndman and Ullah 2007). HU_R_ is proposed to give a better fit and optimistic forecast in the presence of outliers during fitting period, which is very common for all these countries. For illustration, the observed female mortality rates for Russia and Ukraine are given in Fig. 1 for the fitting period.

Although HU_W_ produced an optimistic forecast for Belarus and Ukraine, still the results are the subject to analyze. HU_W_ is employed for countries with long time series data, which was not the case for these countries. Similarly, despite providing more optimistic forecasts and greater forecast accuracy than the other methods, forecasts using HU_R_ are affected by unusual improvements in age-specific mortality patterns more than forecasts using the other two HU variants (see the country-specific example of Hungary for more details). It should be noted that although we tried to utilize all available data from the HMD, it was not possible for all these countries. Due to caution notes from the HMD, we started the fitting period only from the best available years mentioned in the HMD (Table 1), making data quality a restriction for the application of the models. BMS also has some flaws regardless of providing the optimistic forecast for the Baltic counties. BMS considers only the best fitting period instead of taking into account all the observed data. For all nine countries, the data of the last 20 years was found to be more significant for forecasting during model fitting.

To determine the forecast accuracy of the methods (except for the UN forecast), we analyzed the MAE and the MSE during the out-of-sample evaluation period. One-step-ahead MAE and MSE for the LC and HU variants are given in Tables 3 and 4, respectively. As mentioned previously, the models failing to forecast *e*_0_ higher than the last observed *e*_0_ of corresponding fitting periods (Table 2) are omitted. In terms of MAE and MSE, the lowest errors were found for HU_W_. For high-mortality contexts, the lowest MAE and MSE were not always obtained using the identical method for a country. Except for HU variants, all other models overestimated the mortality rates and produced lower life expectancy as a consequence. The other LC variants slightly underestimated the mortality rates during the fitting period. The basic LC method returned a high MAE for Estonia and Lithuania. Some values of MSE indicate overfitting of corresponding models. Forecast accuracies were close for the basic LC variants, except for BMS. Contrary to the optimistic forecast obtained for all three Baltic countries and Slovakia, BMS was not the best forecast technique in term of MAE. The different results of the LC variants imply the influence of different adjusting techniques for the time component of the model. We employed the LC method without any adjustment for the time component, and the results were different than those of the existing LC variants. Nevertheless, in terms of forecast accuracy or an optimistic forecast, it was not possible to declare a particular model unquestionably and uniquely best for all of these countries. For the developed countries, almost the same situations were observed, prompting the conclusion that no model performed uniquely well for all countries (Shang et al. 2011; Shang 2012).

**Table 3:** One-step-ahead forecast bias of the fitted models in terms of MAE

Country	LC	LC_P_	LM	BMS	HU	HU_R_	HU_W_	LL
Belarus	–	–	0.144	–	–	–	0.102	–
Bulgaria	0.160	0.144	0.128	0.122	0.137	0.131	0.110	0.131
Estonia	0.248	0.246	0.322	0.259	0.324	0.292	0.273	0.267
Hungary	0.181	0.164	0.146	0.132	0.119	0.118	0.115	0.157
Latvia	0.186	0.185	0.204	0.186	0.174	0.196	0.175	0.146
Lithuania	0.330	0.167	0.209	0.187	0.167	0.177	0.168	0.210
Russia	–	–	–	–	–	0.079	–	–
Slovakia	0.181	0.177	0.257	0.186	0.200	0.212	0.195	0.214
Ukraine	–	–	–	–	–	0.087	0.066	–

A blank place means the forecast of *e*_0_ was lower than the last observed value of *e*_0_.

**Table 4:** One-step-ahead forecast bias of the fitted models in terms of MSE

Country	LC	LC_P_	LM	BMS	HU	HU_R_	HU_W_	LL
Belarus	–	–	0.079	–	–	–	0.033	–
Bulgaria	0.044	0.037	0.033	0.038	0.047	0.045	0.034	0.034
Estonia	0.141	0.137	0.283	0.151	0.214	0.204	0.176	0.167
Hungary	0.065	0.057	0.049	0.052	0.048	0.047	0.042	0.057
Latvia	0.110	0.110	0.140	0.109	0.115	0.134	0.107	0.051
Lithuania	0.262	0.079	0.128	0.096	0.074	0.086	0.079	0.127
Russia	–	–	–	–	–	0.010	–	–
Slovakia	0.112	0.111	0.1967	0.130	0.169	0.163	0.162	0.109
Ukraine	–	–	–	–	–	0.015	0.008	–

A blank place means the forecast of *e*_0_ was lower than the last observed value of *e*_0_.

#### Coherent forecasting

Our study is unique compared to previous comparisons because we considered coherent mortality forecasting for high-mortality countries as well. Moreover, the reference population of the coherent forecast was completely comprised of countries with high-mortality regime compared to previous studies with mixture of countries from low mortality regimes (Li and Lee 2005). We fitted the model from 1970 to 2010 for coherent forecasting due to data problems in earlier years and ended with Bulgarian mortality data for 2010. Therefore, the 40 years ahead forecasts give us future life expectancy in 2050. The coherent mortality forecast could not produce more optimistic results than the LC and HU variants for any of the countries. However, it was the most accurate method for Latvia in terms of MAE and MSE and for Slovakia in terms of MSE. The scope of coherent mortality forecasting for these comparatively high-mortality countries became restricted due to the lack of long time series data. The results of coherent mortality forecasting for theses countries are shown in Table 2. LL produced the most pessimistic forecast among all methods for Bulgaria, Hungary, Latvia, and Slovakia. For the sake of comparability, the same fitting period (1970–2010) is used for different LC and HU variants, the results are shown in Table 5.

**Table 5:** Comparison of 40 years ahead life expectancy forecast for selected countries with high-mortality for the harmonized fitting period (1970–2010)

Country	*e*_0_	LC	LC_P_	LM	BMS	HU	HU_R_	HU_W_	LL
Belarus	76.49	–	–	–	–	–	–	78.58	–
Bulgaria	77.25	79.92	80.09	79.95	80.09	–	79.17	77.43	77.55
Estonia	80.55	84.73	84.66	84.82	88.23	–	–	80.57	84.71
Hungary	78.34	82.30	82.42	82.78	84.79	85.71	86.42	86.44	82.60
Latvia	77.39	80.28	79.60	79.70	79.75	78.62	-	79.83	80.10
Lithuania	78.73	80.96	79.76	80.52	79.66	79.41	78.96	81.45	81.63
Russia	74.86	–	–	–	–	–	–	74.89	–
Slovakia	79.15	83.50	83.45	83.49	84.67	82.69	82.67	83.45	82.37
Ukraine	75.19	–	–	–	–	–	–	78.90	–

Results are shown for females only.

*e*_0_ is life expectancy at birth of 2010 from the HMD.

A blank place means 40-years-ahead forecasts of *e*_0_ was lower than the *e*_0_ of 2010.

The performance of the coherent mortality forecast was greatly affected for three reasons: (i) combination of mortality rates of a population with large exposure to mortality rates of a population with comparatively smaller exposure size, (ii) high adult male mortality for several of these countries, and (iii) irregular trend of life expectancy of joint mortality data because of (i) and (ii). Although LL performed reasonably for most of these high-mortality countries, it failed to do so for Belarus, Russia, and Ukraine. During estimation of the common factor, countries with high mortality dominated the comparatively low-mortality countries. This might be due to mixing large exposure with smaller exposure or combining mortality rates of populations with different age- and cause-specific mortality patterns. Because country-level mortality has two parts—common factor from the reference population and country-specific estimate of *b*_*x*,*i*_*k*_*t*,*i*_—the country-specific forecast is affected because the common factor of the reference population greatly affects completely different mortality patterns. This is another consequence of using coherent forecasting for population groups with increasing mortality differences over time (mortality divergence). LL adjusts the time component of the common factor according to the estimated life expectancy of combined mortality data. The estimated life expectancy for our high mortality countries has irregular trend that is different than the country-specific individual trends of life expectancy in most of the cases. Characteristics of the combined mortality rates of these countries and the key outcomes of the fitted model using joint mortality rates are given in the Appendix.

Our coherent forecasting results also indicates necessity of implying a rigid assumption for choosing a similar group of countries (Kjærgaard et al. 2016). The six countries for which the coherent mortality forecast of life expectancies is higher than the last observed life expectancies are all currently member states of the European Union. In the current methodology of coherent forecasting, the fitting period of combined mortality data might be shorter for individual countries with longer time series data; this may affect the forecast as well.

We re-discovered the problem of a shorter fitting period for all LC and HU variants during our analysis of the forecast with a harmonized, shortened fitting period. In addition to the coherent mortality forecasting, previous methods also suffered for shortened fitting periods. When using the harmonized fitting period for Bulgaria and Estonia, the HU model failed to produce a higher forecast than the last observed life expectancy, HU_R_ failed to do so for Estonia, Latvia, and Russia. It was mentioned in earlier studies that long time series is preferable for the fitting of these models, however, that condition could not be held for many of these countries due to data problems (Booth et al. 2002). As the HMD mentioned, there was lower data quality for some of the years for a few of these countries due to the data source; we tried to fit the models by both including and excluding those years. The forecasts obtained by considering those years during the fitting period were misleading. However, we could not fit the models for Estonia and Lithuania by excluding the problematic years, as they are almost in the middle of the fitting period for Estonia, and exclusion could make the fitting period too short for the forecasts in the case of Lithuania. In several cases, particular models failed to produce higher estimates of life expectancies than the last observed one; estimates obtained considering all available data for model fitting differed. A comparison of one-step-ahead MAE and MSE for the LC and HU variants and LL are given in Tables 6 and 7 for the harmonized fitting period.

**Table 6:** One-step-ahead forecast bias of the fitted models in terms of MAE considering harmonized fitting period

Country	LC	LC_P_	LM	BMS	HU	HU_R_	HU_W_	LL
Belarus	–	–	–	–	–	–	0.113	–
Bulgaria	0.120	0.119	0.120	0.118	-	0.119	0.151	0.131
Estonia	0.348	0.289	0.327	0.293	-	-	0.235	0.267
Hungary	0.130	0.182	0.184	0.148	0.139	0.172	0.115	0.157
Latvia	0.337	0.185	0.139	0.138	0.114	0.121	0.112	0.146
Lithuania	0.287	0.209	0.395	0.207	0.208	0.209	0.190	0.210
Russia	–	–	–	–	–	–	0.046	–
Slovakia	0.142	0.142	0.142	0.153	0.163	0.156	0.149	0.214
Ukraine	–	–	–	–	–	–	0.061	–

A blank place means the corresponding 40-years-ahead forecast of *e*_0_ was lower than the *e*_0_ of 2010.

**Table 7:** One-step-ahead forecast bias of the fitted models in terms of MSE considering harmonized fitting period

Country	LC	LC_P_	LM	BMS	HU	HU_R_	HU_W_	LL
Belarus	–	–	–	–	–	–	0.037	–
Bulgaria	0.039	0.039	0.039	0.038	–	0.039	0.062	0.034
Estonia	0.320	0.197	0.283	0.194	–	–	0.122	0.167
Hungary	0.067	0.063	0.072	0.056	0.046	0.059	0.040	0.057
Latvia	0.075	0.040	0.044	0.042	0.031	0.033	0.028	0.051
Lithuania	0.227	0.119	0.307	0.114	0.141	0.136	0.124	0.127
Russia	–	–	–	–	–	–	0.003	–
Slovakia	0.041	0.040	0.042	0.049	0.062	0.060	0.059	0.109
Ukraine	–	–	–	–	–	–	0.007	–

A blank place means the corresponding 40-years-ahead forecast of *e*_0_ was lower than the *e*_0_ of 2010.

#### UN forecasting

Unlike most of the previous comparisons, we extend the comparison of the mortality forecasting models with probabilistic forecasting obtained through a Bayesian framework (UN forecast). It should be noted that the UN life expectancy forecasts shown in Table 2 refer to years 2050–2055, as this technique uses 5 years age groups. For the nine countries considered in this study, we utilized the life expectancy at birth in 5-year intervals from the HMD instead of using the UN data. We project the life expectancy at birth up to year 2100, to compare with the LC and HU variants, the results are shown up to 40 years. In addition to the data from HMD, we also used the data from the World Population Prospects (UN 2013); the comparison between these two different data sources is given in the Appendix. The simulation was done with 160,000 iterations (10,000 burn-in); the thinning interval was 10, and the number of chains was three. It was already mentioned in the previous section that an irregular trend in mortality is visible in the case of several of these countries. The trend of life expectancies computed on a 5-year basis was also similar to that; fluctuations in the trend for life expectancies remained for Belarus, Russia, and Ukraine during the fitting period. Like HMD, a similar pattern was also observed in case of data from World Population Prospects. Nevertheless, UN forecasts showed an increasing trend for life expectancy at birth for all these countries (both for HMD and World Population Prospects). Unlike several LC variants and coherent forecasting, the forecast of life expectancies was higher than the last observed one for all three high-mortality countries—Belarus, Russia, and Ukraine. For all other countries, the forecast produced using the UN forecast technique fell between the other forecast methods. The UN forecast was lowest for Hungary and Latvia among all the methods.

One shortcoming of the UN forecasting technique is that it is not based on life table like the previous LC or HU variants. This prevents age-specific mortality forecasting and comparing forecast accuracy in out-of-sample evaluation period, as we have done for the LC and HU variants or for the coherent forecasting. Instead, we plotted the 95% prediction interval to forecast life expectancy at birth for all nine countries (see Appendix). These prediction intervals are showed here over the whole projection period (up to year 2100). Belarus, Russia, and Ukraine have wider prediction intervals compared to the other countries. The diverging mortality pattern affects the forecast of life expectancy as well.

### Country-specific illustration: Hungary

#### Past mortality trends

**Fig. 2:**
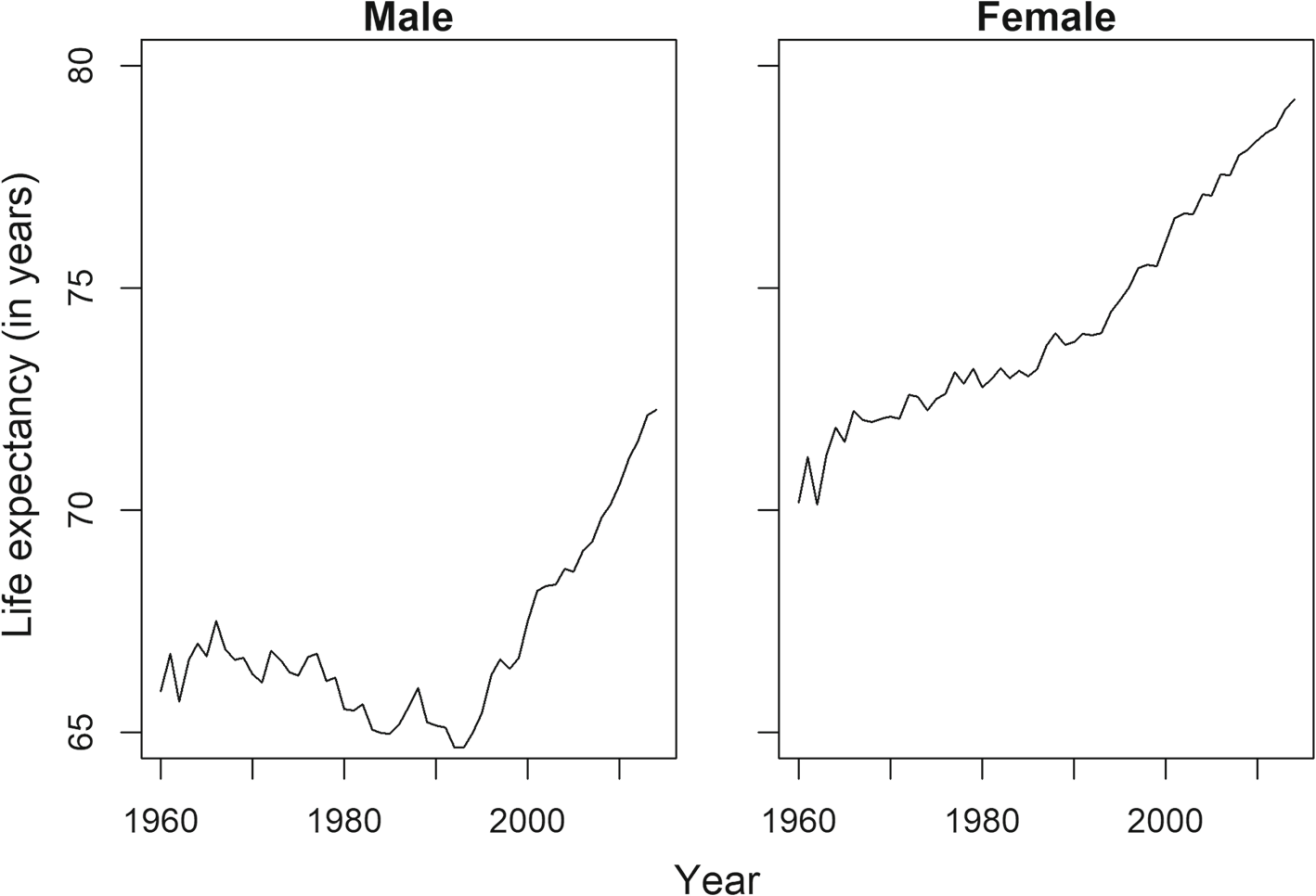
Observed life expectancy at birth of Hungary (1960–2014)

We assessed all the models in the previous section without focusing on a specific country to get an overall view of mortality forecasting in a high-mortality context. To illustrate the performance of different mortality forecasting models in a more detailed way, Hungary is considered in this section as a representative of these countries. Hungary is chosen because it has a high-mortality regime similar to many other countries in Eastern Europe. The common features of the mortality scenario of these countries can be characterized by the presence of a high level of mortality from cardiovascular diseases and several external causes of deaths (Bálint and Kovács 2015). The trend of life expectancy at birth of Hungary is plotted in Fig. 2.

**Fig. 3:**
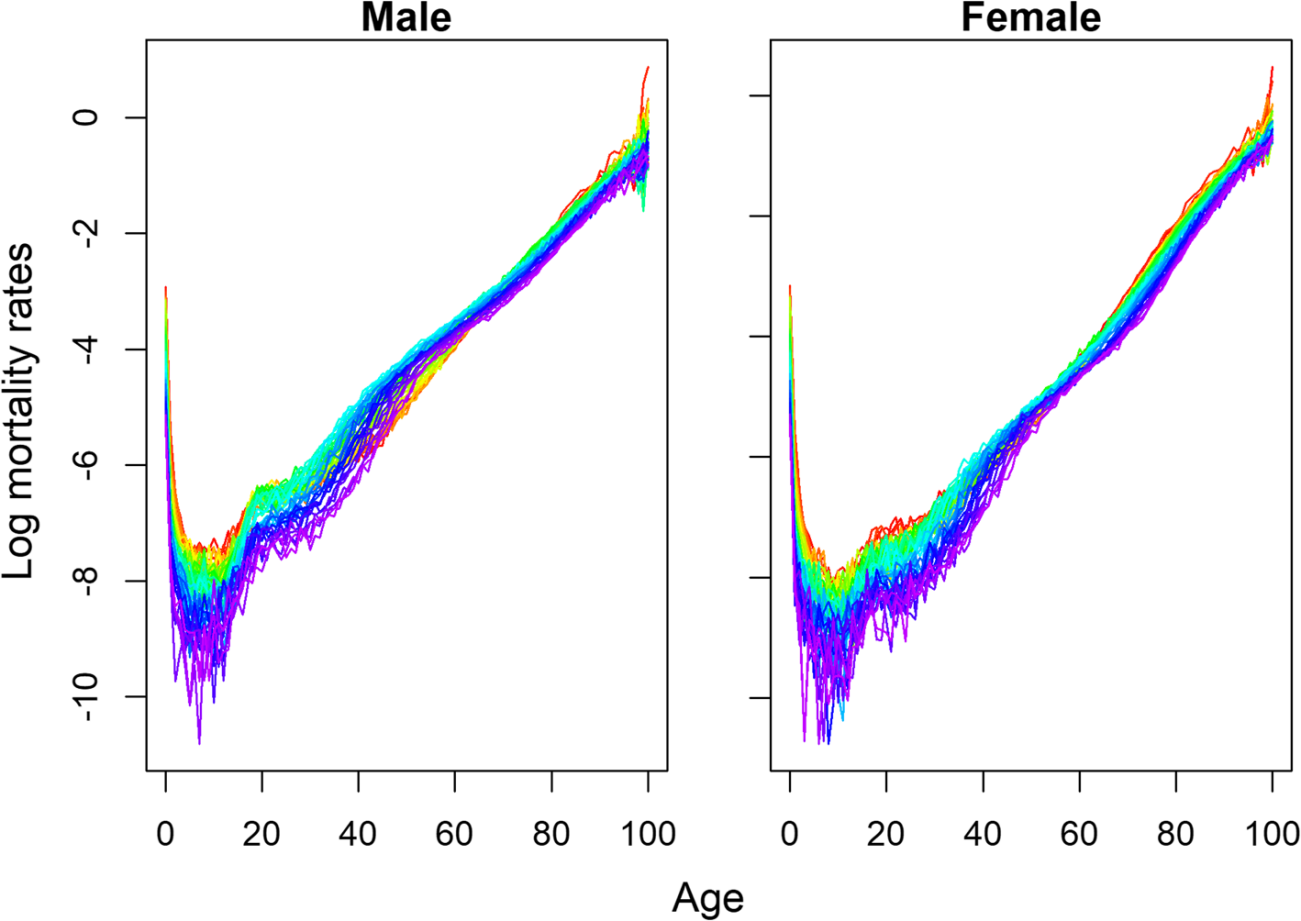
Observed log mortality rates of Hungary (1960–2014). Years are plotted using a rainbow palette as Fig. 1

In the beginning of the 1990s, life expectancy of the Hungarian population was among the lowest in Europe. The recent gains in longevity are related to specific causes of death. Decomposition of life expectancy at birth showed that the 7-year gain in male life expectancy between 1990 and 2013 was mainly attributable to a decline in cardiovascular mortality, which corresponds to 40% of the total gain in longevity (Bálint and Kovács 2015). The decline in mortality due to external causes of deaths has resulted in an increase in life expectancy of 1.7 years. The log mortality rates for Hungarian males and females from the HMD are plotted in Fig. 3. The irregular patterns in mortality are visible in the young and senescence age groups. A gender gap is also visible in different age groups during the fitting period.

#### Forecast of life expectancies

To compare the different methods more precisely in a high-mortality context, we compared the forecasts for all the LC and HU variants and coherent forecasting for males and females separately for Hungary. The results are given in Table 8. We extrapolated the results for 20, 30, and 40 years ahead (24, 34, 44 years for LL) to see any possible convergence of the forecasts using different methods. Life expectancy at birth was 72.26 and 79.24 years respectively for males and females in 2014, while for coherent forecasting, it was 70.59 and 78.34 years, respectively, in 2010. Except for LC, L*C*_*P*_, and BMS, all the models produced a higher forecast of life expectancy than the last observed *e*_0_ of Hungarian males. For females, all the models produced an optimistic forecast. We also forecast remaining life expectancy at age 65 to see the performance of the life expectancy forecasting in later ages and extrapolated for 20, 30, and 40 years ahead (Table 9). Remaining life expectancy at age 65 was 14.56 years and 18.40 years, respectively, for males and female in 2014, while it was 13.94 years and 17.88 years, respectively, in 2010 (for coherent forecasting).

**Table 8:** Forecast of life expectancies at birth for Hungarian males and females

Forecast period	LC	LC_P_	LM	BMS	HU	HU_R_	HU_W_	LL
Male, 2034	–	–	73.93	–	77.35	77.53	77.96	73.19
Female, 2034	81.17	81.06	81.58	82.90	82.22	80.85	83.86	80.96
Male, 2044	–	–	74.42	–	79.33	79.70	80.42	74.23
Female, 2044	82.18	82.07	82.65	84.50	83.47	81.94	85.91	82.00
Male, 2054	–	–	74.82	–	81.03	81.63	82.97	75.24
Female, 2054	83.16	83.05	83.68	85.98	84.64	82.99	87.81	83.01

A blank place place means the forecast of *e*_0_ was lower than the observed value of *e*_0_ for 2014 (2010 for LL)

**Table 9:** Forecast of remaining life expectancies at age 65 for Hungarian males and females

Forecast period	LC	LC_P_	LM	BMS	HU	HU_R_	HU_W_	LL
Male, 2034	21.04	–	17.14	14.77	16.65	17.29	17.42	15.21
Female, 2034	20.29	20.19	20.26	20.93	20.59	19.35	21.72	19.49
Male, 2044	22.84	–	18.61	15.26	17.71	18.49	19.10	15.77
Female, 2044	21.15	21.06	21.18	22.12	21.52	20.07	23.35	20.17
Male, 2054	25.02	–	20.27	15.76	18.73	19.67	21.10	16.35
Female, 2054	22.00	21.91	22.08	23.27	22.41	20.82	24.90	20.87

A blank place means the forecast of *e*_65_ was lower than the observed value of *e*_65_ for 2014 (2010 for LL)

Although LC and BMS failed to produce optimistic forecasts of life expectancies at birth, the forecast of *e*_65_ was higher than the last observed *e*_65_ for both methods; which was not the case for L*C*_*P*_. In the other cases, all the models performed well, although higher forecasts are observed from HU_W_ for females compared to the other methods. Moderate improvements in the mortality of people aged 65 years or over were observed in previous studies as well, unlike the pattern observed for the middle-aged population (Bálint and Kovács 2015).

#### Forecast of mortality rates

**Fig. 4:**
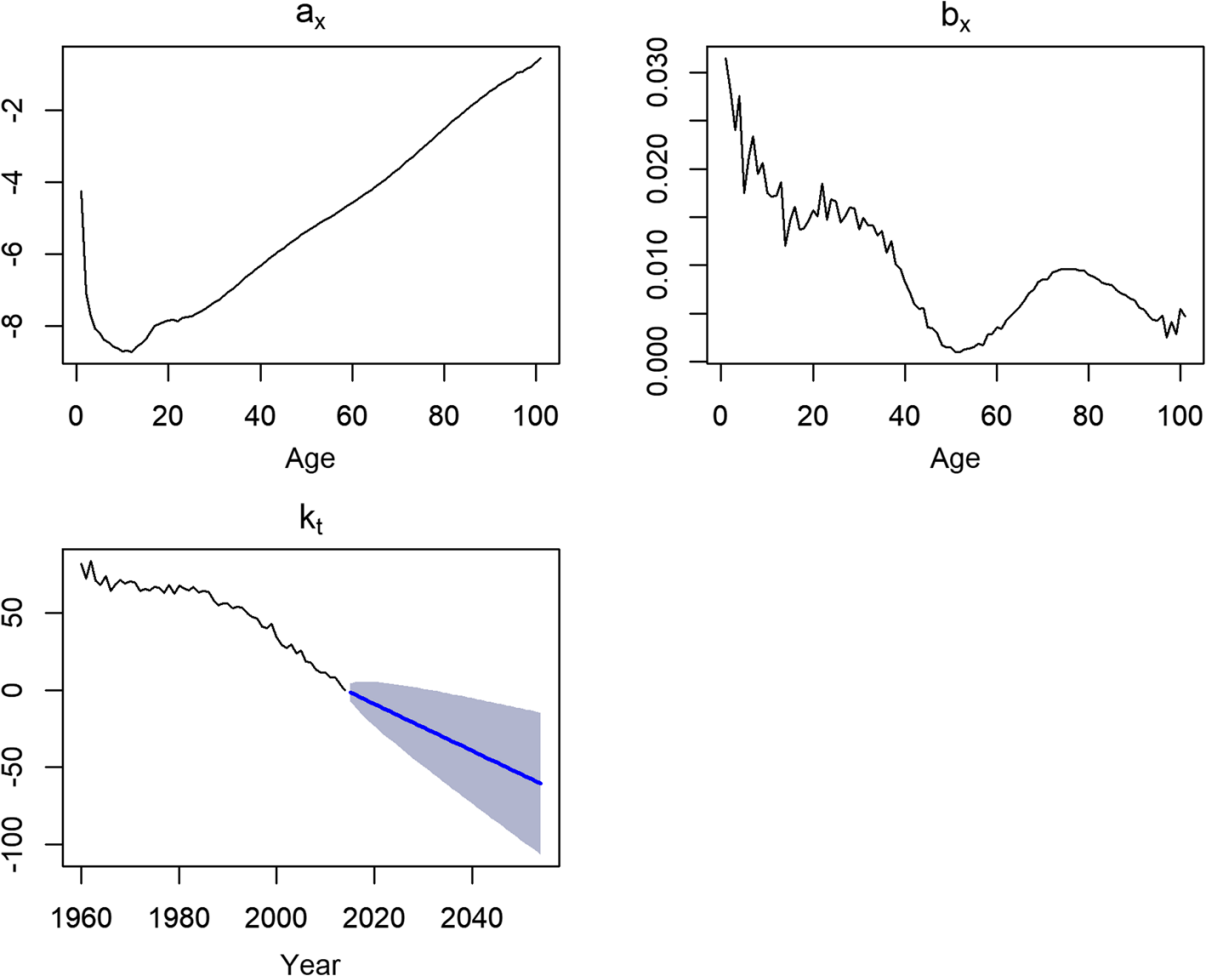
Fitted components of basic LC method for females of Hungary (1960:2014). The blue area of the parameter *k*_*t*_ presents spread of the parameter under a random walk with drift which is used to make forecast

**Fig. 5:**
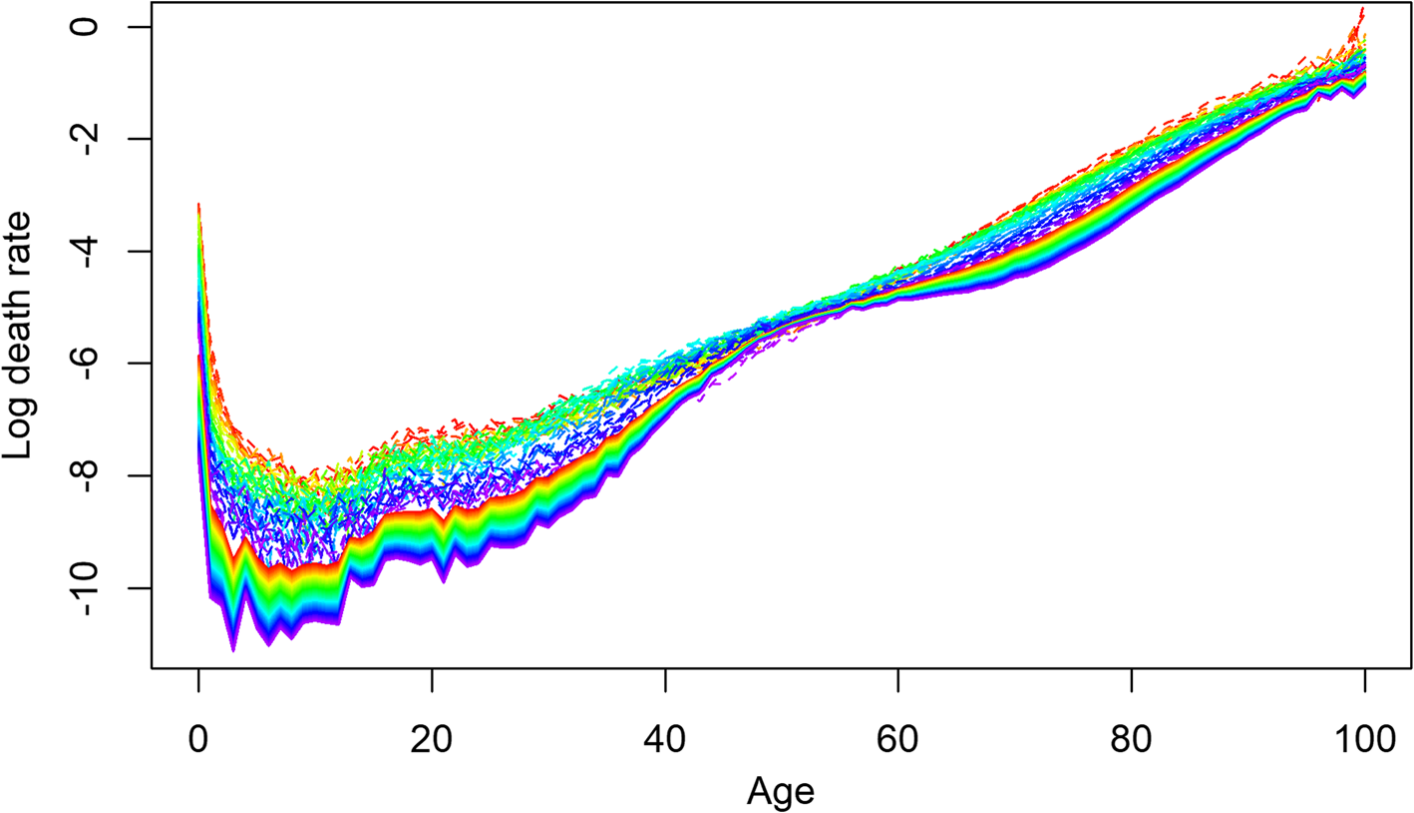
Observed (1960:2014) and 40 years ahead forecast (2015:2054) of log mortality rates for females of Hungary by basic LC method. Years are plotted using a rainbow palette as before. Observed mortality rates are plotted using dotted line whereas forecast are plotted with regular line. Due to mortality improvement, the forecasts of mortality rates are below the observed mortality curves and more close to each other

Besides optimistic forecast of life expectancies and accuracy of during out-of-sample evaluation period, we examined more deeply the ability of the methods to capture the mortality improvement over the life span in this section. Moreover, we observed that some methods have different performances for different stages of lifespan (Tables 8 and 9). The fitted parameters of the LC model with a forecast of parameter *k*_*t*_ and the observed and fitted log mortality rates for females are presented in Figs. 4 and 5. As discussed before, the parameter *k*_*t*_ is used for the forecast; the blue spread of *k*_*t*_ in Fig. 4 may be implemented for interval forecasting as well (Hyndman and Ullah 2007). The main source of gain in female life expectancy was the result of the decline in adult and senescence mortality for some particular causes of deaths. Previous studies revealed the contribution of the female population aged 64 years and over was more substantial on increasing life expectancy than that of middle-aged females those were also affected by the economic crisis in past (Bálint and Kovács 2015; Bohk and Rau 2015).

**Fig. 6:**
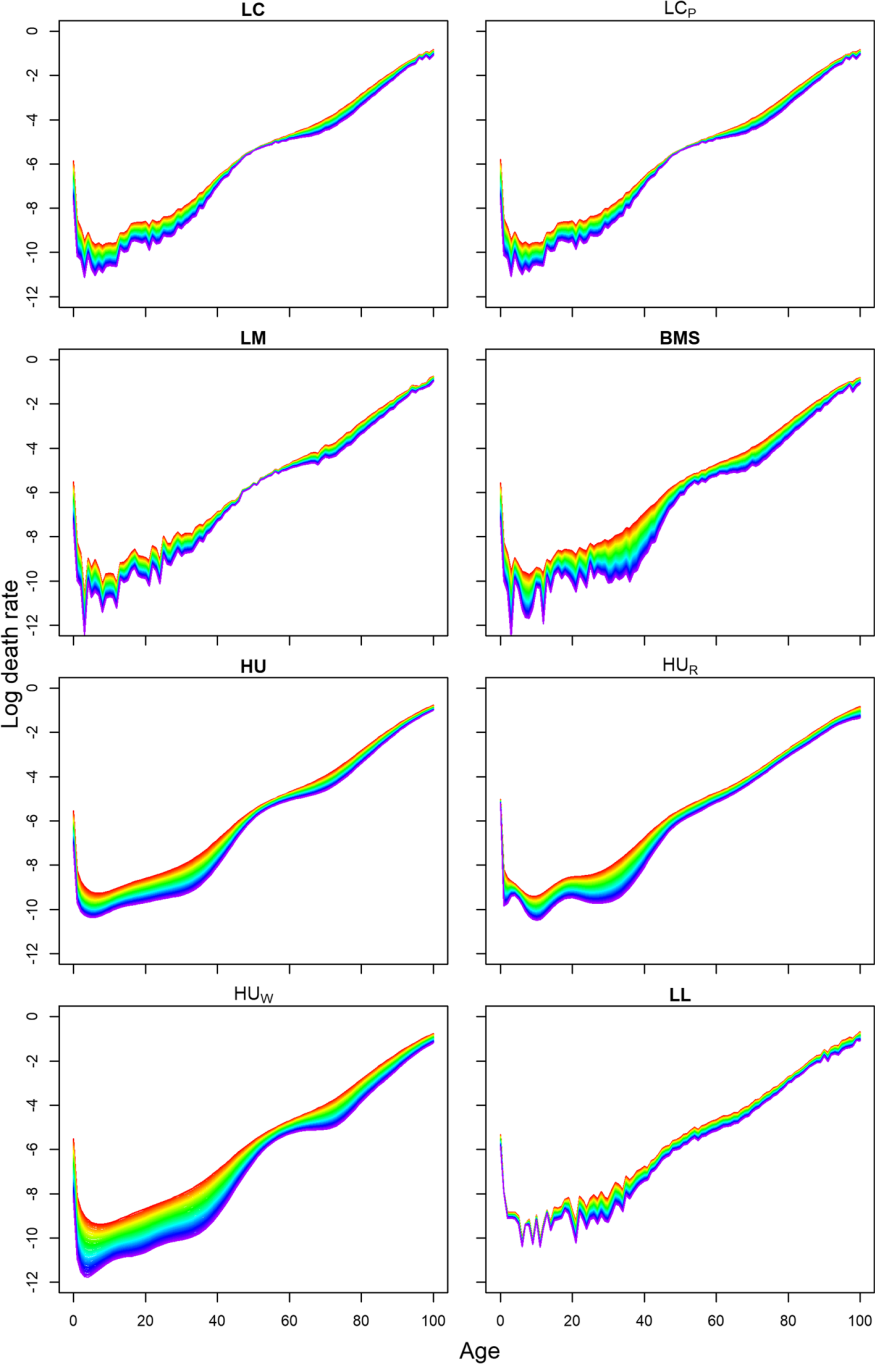
Comparison of 40 years ahead forecast of log mortality rates for females of Hungary. Years are plotted using a rainbow palette

We already compared the forecasts for life expectancies at different ages. Figure 6 shows the 40-years-ahead forecast of log mortality rates for Hungarian females using different methods.

Here, years are plotted again using a rainbow palette, so the beginning years of the fitting period are in red, followed by orange, yellow, green, blue, and indigo, with the most recent years plotted in violet. All the models forecast a rapid and sharp decline in infant and adult mortality, except for coherent forecasting (Fig. 6). Except for HU and HU_W_, all other methods are affected by the recent short-run improvement of early-aged mortality around age 5 to 15. For Hungarian female mortality, this improvement occurred only for a few of the very recent years (Fig. 3) which highly affects the forecasts of BMS, HU_R_, and possibly LL. Combined mortality rates of the reference population were higher across the life span compares to observed female mortality rates for Hungary. As a consequence, the coherent forecasts for the Hungarian females are more pessimistic than those of the other methods (Tables 8 and 9). We already mentioned it as a drawback of current settings for choosing reference population in coherent forecasting. Clearly, the increasing mortality differences over time between the countries of the reference group have high impact on individual country-specific forecasts. Forecasts of senescence mortality are virtually similar for all methods except for coherent forecasting due to this reason.

## Conclusion and discussion

### Summary of results

We compared and contrasted mortality forecasting models for higher mortality regimes that lack long time series data of good quality, which is common in several Central and Eastern European (CEE) countries. The performance of the forecasting models for the nine CEE countries was found to be lower than that observed for low-mortality countries. No model gives uniquely best performance for all the nine CEE countries included. All studied LC variants, with the exception of the weighted version of the (Hyndman and Ullah 2007) method, resulted in lower future life expectancy than current observed life expectancy values for Belarus, Russia, and Ukraine. Coherent mortality forecasting could not overcome the limitations of the LC variants completely by producing more optimistic forecasts or more accurate forecasts during out-of-sample evaluation. The UN forecasting technique (through a Bayesian hierarchical model) can provide a more optimistic forecast than some LC variants for Belarus, Russia, and Ukraine, but it also produced a wider prediction interval for several of these countries. The country-specific forecast for Hungary revealed that some of the models can overcome the shortcomings of pessimistic forecasts of the earlier part of the life span.

### Explanations of the main findings

The performance of the forecasting models for the nine CEE countries was found to be lower than that of the observed for low-mortality countries. In addition to a high-mortality regime, the lower performance of the forecasting models for the nine CEE countries is highly attributable to the irregular mortality trends and a shorter fitting period. Except for weighted and robust variants of the HU method, most of the LC variants were not appropriate for some of the high-mortality countries. The mentioned two methods succeed to produce optimistic forecast for these countries due to application of different weighting techniques, which was not the case for the other methods. Except for Hungary and Slovakia, all of these countries suffered from mortality crisis during 1990s, and it was particularly severe for Belarus, Russia, and Ukraine (Shkolnikov et al. 1998). The accuracy obtained for weighted HU method during out-of-sample evaluation is thus subject to analyze as this method gives more weight on recent period than distant past (Hyndman and Ullah 2007).

Coherent mortality forecasting could not overcome the limitations of the LC variants completely by producing more optimistic forecasts or more accurate forecasts during out-of-sample evaluation. This might be explained by the high heterogeneity across CEE countries in terms of mortality levels and patterns. Such heterogeneity increased during the severe mortality crisis which Belarus, Russia, and Ukraine underwent during the 1990s (Shkolnikov et al. 1998). This lead to pessimistic forecasts even by coherent model.

In this case, UN forecast (using a Bayesian hierarchical model) can provide more optimistic forecasts: life expectancy at bith rather age-specific rates are considered, and in this way, the cross-country differences are mitigated. However, due to irregular trends of life expectancies over time, UN forecasts also produced wider prediction intervals for several of these countries.

The country-specific forecast for Hungary revealed that some of the models can overcome the shortcomings of pessimistic forecasts of life expectancy in earlier part of life. For three LC variants, we observed that the models produce optimistic forecast of life expectancy at later ages despite of failing to do so for life expectancy at birth for Hungarian male.

### Limitations

The current study have several limitations. Firstly, we could not consider all possible mortality data. With only nine high-mortality CEE countries we just have a snapshot of the limitations of the mortality forecasting models from a different point of view. This selection was necessary in order to keep data quality at the highest level. Moreover, we did not consider data of Poland or the Czech Republic as their mortality patterns are more similar to low-mortality countries. Secondly, we did not utilize all types of existing mortality forecasting models. For example, we did not consider behavioral impacts on mortality forecast. Impact of smoking and alcohol consumption on life expectancy are mentioned in previous studies (see Luy and Wegner-Siegmundt 2014; Trias-Llimós et al. 2017, for example on smoking and alcohol consumption, respectively). In addition, forecasting considering different causes of deaths could also provide more insight. However, data regarding causes of death or risk factors are not available for some of these countries. Thirdly, we did not compare the interval forecast for LC and HU variants, which could be another topic for further research. Fourthly, due to the unavailability of data for later age groups, we restricted the model fitting to age 100. Such truncation is not suitable for a wide forecast horizon, even for a population with less of an aging problem. Finally, we considered all populations together for coherent forecasting, without following any particular strategies to choose the best reference population (Kjærgaard et al. 2016).

### Recommendations for future work

Based on the study findings, future research directions for mortality forecasting can be suggested. A significant improvement would be to develop a new forecasting technique flexible enough to handle this sort of irregularities in mortality trends as most of the LC variants could not perform well in the presence of irregular mortality trends. Most of the existing methods for mortality forecasting are based on the LC method to some extent. Better performances of the HU variants can be attributable to smoothing techniques adopted in those methods. Instead of considering only one-dimensional smoothing (smoothing the age-specific mortality rates of a given year), incorporating the period and/or cohort effect may provide different results than those obtained here. Second-level estimation for fitted parameters in the LC method has a great impact on forecast accuracy for some countries; an alternative estimation approach at this level could also be useful. Coherent mortality forecasting clearly indicates a lack of rigid assumptions for choosing an appropriate set of countries as a reference group. In the current methodology, a shortened, harmonized fitting period for a group of countries also reduces the scope of forecasting. New strategies for choosing a reference population need to address the issues of increasing mortality differences between countries over time (mortality divergence). To overcome the lack of mortality data for later age groups (say after age 100), some extrapolation before forecasting might be useful for longer forecasts. Flexible settings in terms of the fitting period for modeling the joint mortality data may improve the forecast accuracy of country-specific forecasts. Nevertheless, the invention of a new forecasting method for high mortality countries is necessary based on the findings of the current study, as none of the models was completely suitable for the examined nine CEE countries.

## Appendix

### Key features of coherent forecasting for high mortality countries

**Fig. 7:**
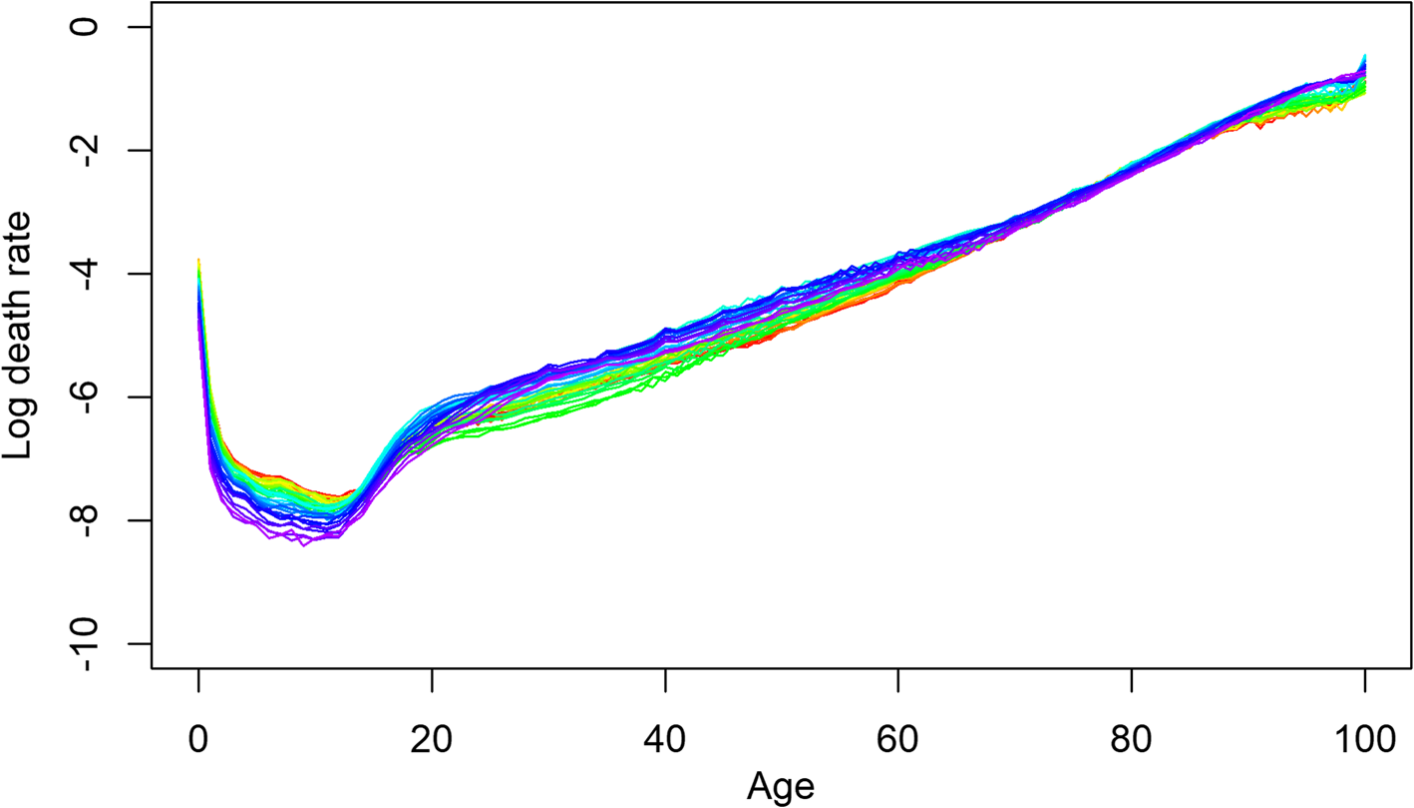
Observed log mortality rates for combined mortality data of (comparatively) high mortality countries. Years are plotted using a rainbow palette so the earlier years are shown in red, followed by orange, yellow, green, blue, and indigo with the most recent years plotted in violet. Mortality crisis in recent period is more visible than previous trend specially for adult and later senescence age groups

**Fig. 8:**
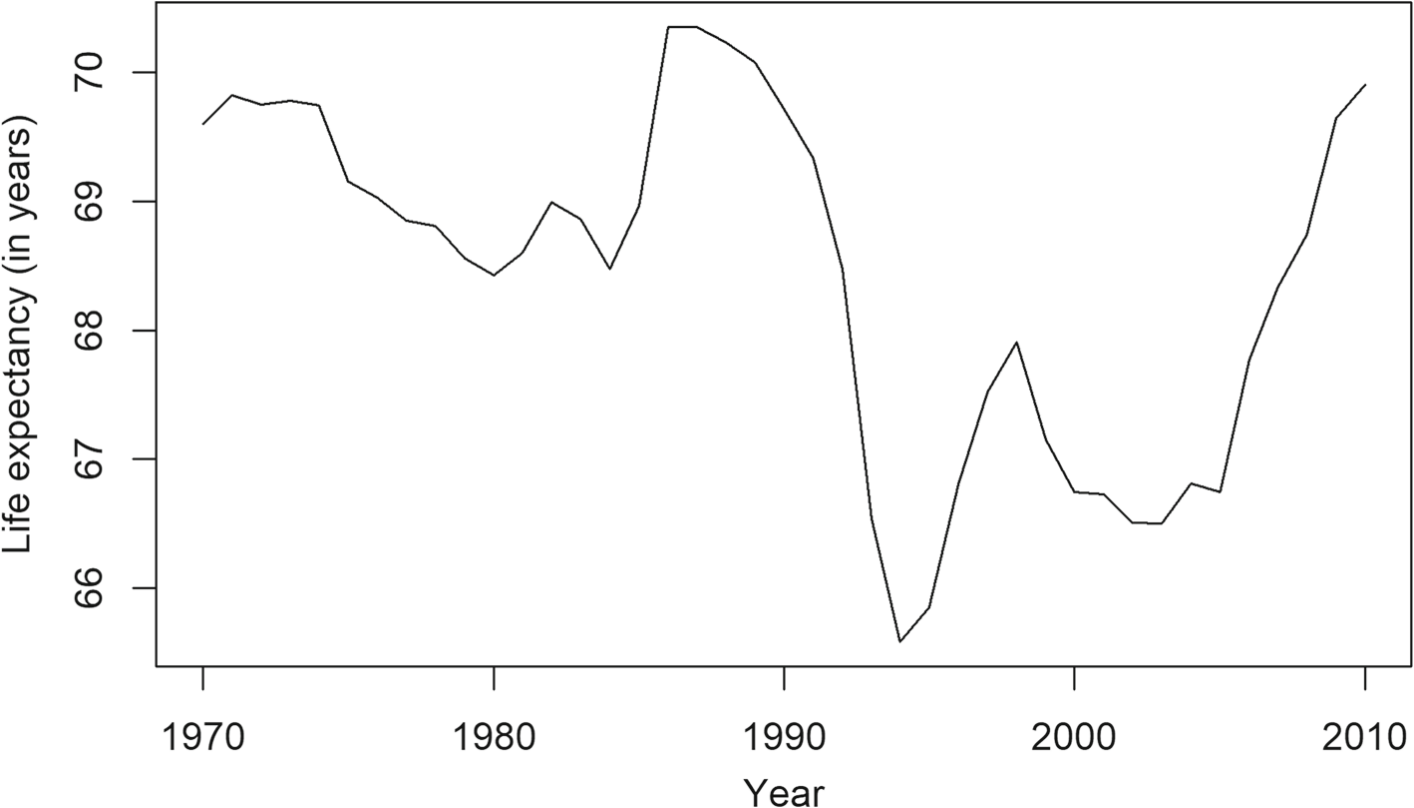
Observed (estimated) life expectancy at birth for combined mortality data of (comparatively) high mortality countries (1970:2010)

### Fitted model on joint mortality rates

**Fig. 9:**
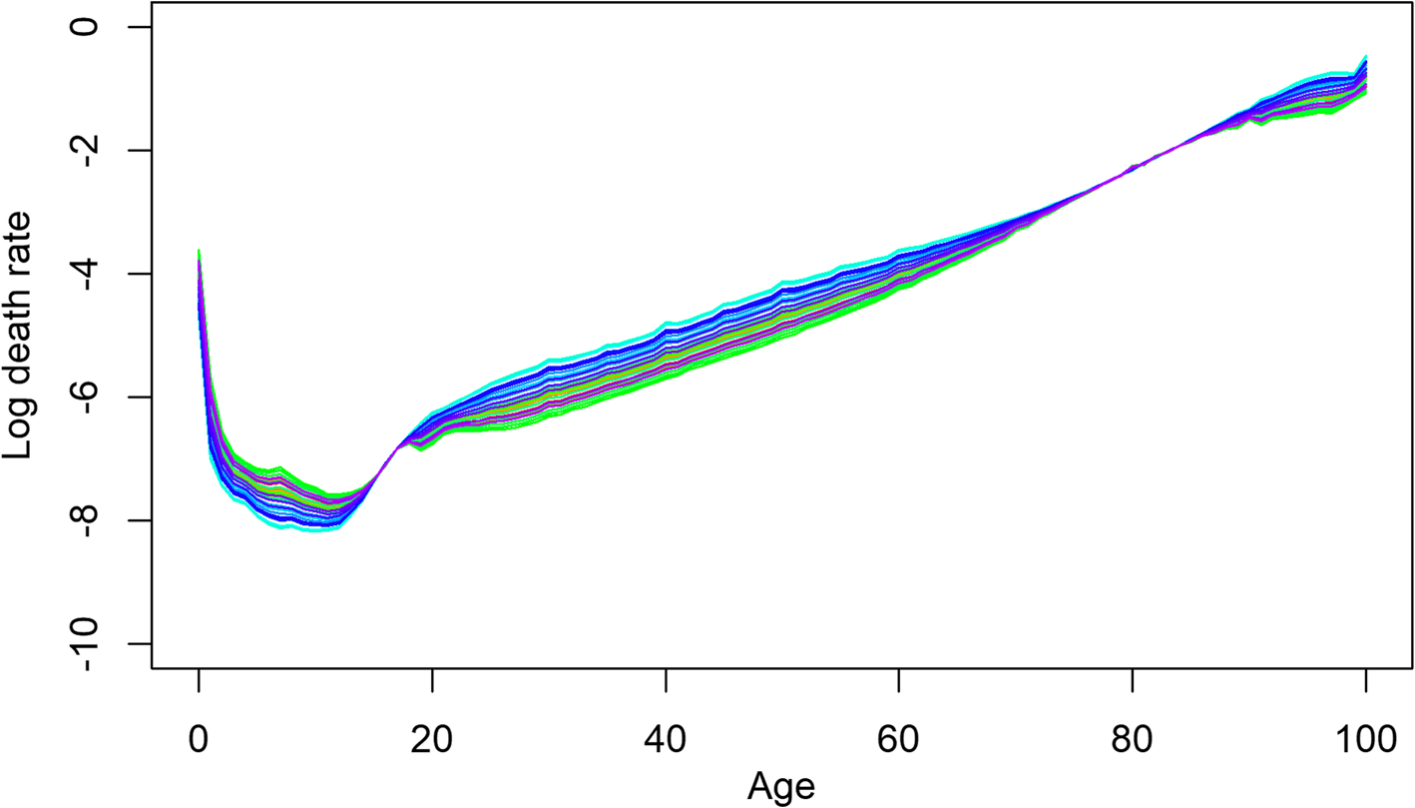
Fitted log mortality rates for combined mortality data of (comparatively) high mortality countries. Years are plotted using a rainbow palette as before

**Fig. 10:**
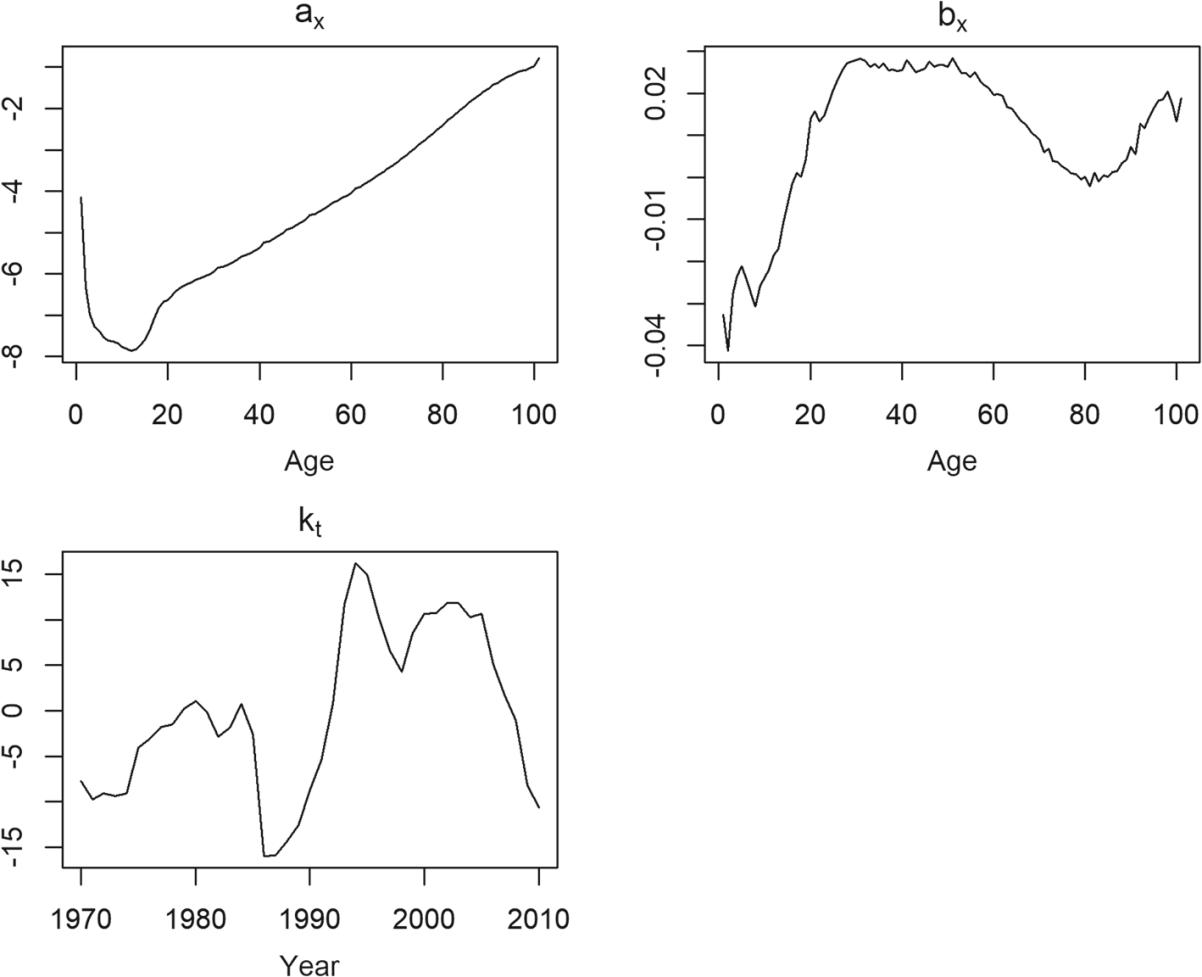
Estimated parameters of first stage LC modeling on joint mortality data. Total 70% variation was explained by the fitted model

### UN forecast for (comparatively) high mortality countries

**Fig. 11:**
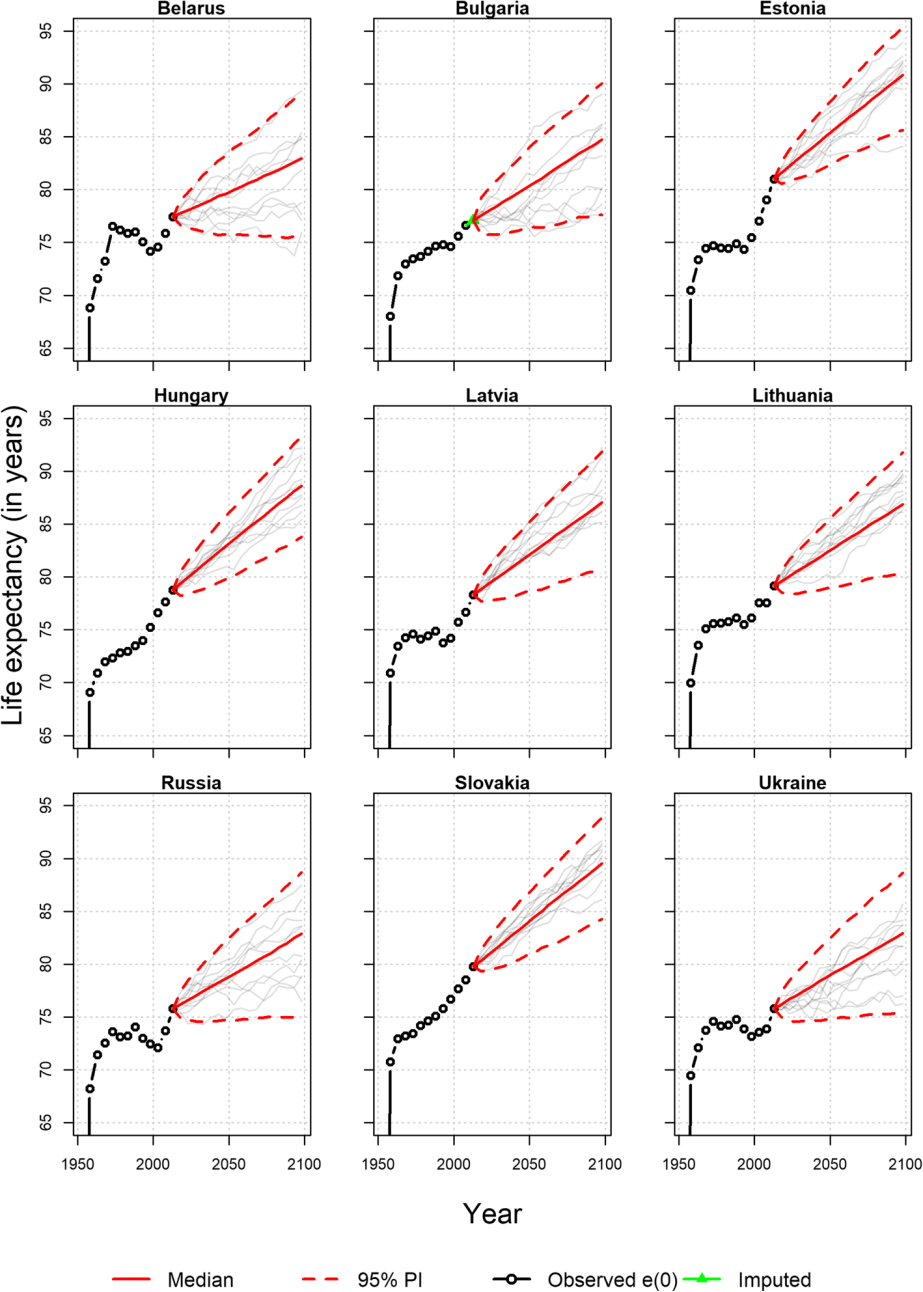
Forecast of life expectancy at birth for (comparatively) high mortality countries by UN forecast with 95% prediction interval. Results are showed for females only

**Table 10:** Forecast of life expectancies at birth using UN forecast method for HMD-2018 and WPP- 2012^*†*^

Data and forecast year	Belarus	Bulgaria	Estonia	Hungary	Latvia	Lithuania	Russia	Slovakia	Ukraine
HMD, 2034	78.672	78.901	83.381	81.165	80.361	80.938	77.463	82.109	77.507
WPP, 2034	77.225	78.914	81.395	80.600	78.960	79.807	76.223	81.071	75.621
HMD, 2044	79.370	79.740	84.528	82.311	81.384	81.851	78.265	83.273	78.371
WPP, 2044	78.022	79.858	82.371	81.592	79.724	80.645	77.204	82.057	76.219
HMD, 2054	79.952	80.633	85.703	83.539	82.432	82.763	79.079	84.436	79.243
WPP, 2054	78.812	80.729	83.315	82.707	80.564	81.428	77.991	83.053	76.906

*†*For WPP-2012 data, the life expectancies were available from 1873 to 2015. The results are shown for females only
